# CONVENTIONAL VIDEOENDOSCOPY CAN IDENTIFY *HELICOBACTER PYLORI* GASTRITIS?

**DOI:** 10.1590/0102-6720201600020002

**Published:** 2016

**Authors:** Alexandre GOMES, Thelma Larocca SKARE, Manoel Alberto PRESTES, Maiza da Silva COSTA, Roberta Dombroski Petisco, Gabriela Piovezani RAMOS

**Affiliations:** 1 Postgraduate Program in Principles of Surgery, Evangelic Faculty of Paraná/University Evangelic Hospital of Curitiba/Medical Research Institute, Curitiba, PR;; 2 Gastrointestinal Endoscopy Service of the 9 of July Hospital), São Paulo, SP, Brazil

**Keywords:** Helicobacter pylori, Gastritis, Endoscopy, gastrointestinal

## Abstract

**Background:**

Studies with latest technologies such as endoscopy with magnification and chromoendoscopy showed that various endoscopic aspects are clearly related to infection by *Helicobacter pylori* (HP). The description of different patterns of erythema in gastric body under magnification of images revived interest in identifying these patterns by standard endoscopy.

**Aim:**

To validate the morphologic features of gastric mucosa related to *H. pylori* infection gastritis allowing predictability of their diagnosis as well as proper targeting biopsies.

**Methods:**

Prospective study of 339 consecutive patients with the standard videoendoscope image analysis were obtained, recorded and stored in a program database. These images were studied with respect to the presence or absence of *H. pylori*, diagnosed by rapid urease test and/or by histological analysis. Were studied: a) normal mucosa appearance; b) mucosal nodularity; c) diffuse nonspecific erythema or redness (with or without edema of folds and exudate) of antrum and body; d) mosaic pattern with focal area of hyperemia; e) erythema in streaks or bands (red streak); f) elevated (raised) erosion; g) flat erosions; h) fundic gland polyps. The main exclusion criteria were the use of drugs, HP pre-treatment and other entities that could affect results.

**Results:**

Applying the exclusion criteria, were included 170 of the 339 patients, of which 52 (30.58%) were positive for HP and 118 negative. On the positive findings, the most associated with infection were: nodularity in the antrum (26.92%); presence of raised erosion (15.38%) and mosaic mucosa in the body (21.15%). On the negative group the normal appearance of the mucosa was 66.94%; erythema in streaks or bands in 9.32%; flat erosions 11.86%; and fundic gland polyps 11.86%.

**Conclusion:**

Endoscopic findings are useful in the predictability of the result and in directing biopsies. The most representative form of HP related gastritis was the nodularity of the antral mucosa. The raised erosion and mucosa in mosaic in the body are suggestive but not specific to the infection. The other forms were not conclusive of the presence of HP.

## RESUMO

**Racional:**

Estudos com tecnologias mais recentes como endoscopia com magnificação e cromoscopia mostraram que vários aspectos endoscópicos estão claramente associados à infecção por *Helicobacter pylori*. A descrição de padrões diferenciados de enantema no corpo gástrico através da magnificação de imagens reavivou o interesse na identificação desses padrões pela endoscopia convencional.

**Objetivo:**

Validar os padrões morfológicos de mucosa gástrica usando videogastroendoscopia convencional relacionados à gastrite por infecção por *Helicobacter pylori*, permitindo previsibilidade do seu diagnóstico e o direcionamento de biópsias.

**Métodos:**

Estudo prospectivo de 339 pacientes consecutivos com análise das imagens de videogastroendoscopia obtidas, gravadas e armazenadas em banco de dados. Estas imagens foram estudadas com relação à presença ou não do *Helicobacter pylori* diagnosticado por teste rápido de urease e/ou por pesquisa direta por estudo anatomopatológico. Foram estudados: a) aspecto normal da mucosa; b) nodularidade da mucosa; c) enantema inespecífico difuso de antro e corpo; d) enantema em mosaico ou salpicado; e) enantema em estrias ou faixas; f) erosões elevadas; g) erosões planas; h) pólipos de glândulas fúndicas. Os principais critérios de exclusão foram o uso de medicamentos, tratamento prévio de HP e outras entidades que pudessem interferir nos resultados.

**Resultados:**

Aplicando os critérios de exclusão, incluíram-se 170 dos 339 pacientes sendo 52 (30,58%) positivos para *Helicobacter pylori* e 118 negativos. No grupo positivo os achados que mais se associaram com a infecção foram: nodularidade no antro (26,92%); presença de erosões elevadas (15,38%) e mucosa em mosaico no corpo (21,15%). No grupo negativo o aspecto normal da mucosa foi de 66,94%; enantema em estrias ou faixas em 9,32%; erosões planas em 11,86%; e pólipos de glândulas fúndicas 11,86%.

**Conclusão:**

Achados endoscópicos são úteis na previsibilidade de localização e direcionamento de biópsias na pesquisa do HP. A mais representativa forma de gastrite por HP foi o achado de nodularidade na mucosa antral. As erosões elevadas e mucosa em mosaico no corpo são sugestivas, mas não específicas da infecção. As demais formas não foram conclusivas da presença do HP.

## INTRODUCTION

Since the discovery of Helicobacter pylori (HP) in 1983, strong evidences have indicated that the infection has an important role in the pathogenesis of chronic gastritis, peptic ulcer and gastric cancer^16^. Gastritis involves cell damage, regenerative and inflammation of the mucosa, with presence of lymphoid follicles. The inflammatory process is initially superficial but, in sequence, affects the entire mucosa, first in the antrum and progressing proximally to the body. Over the years the gastric glands are destroyed, showing epithelial atrophy and intestinal metaplasia areas that favor the appearance of gastric carcinoma^23^.

The diagnosis of the infection requires at least two tests in accordance with the european guidelines^22^. The most used are the rapid urease test and histological analysis^25^. The rapid urease test has a sensitivity of 92% and specificity of 95%^5^. Laine et al.^18^ found histological sensitivity variation in the identification of HP bacteria according to the bacterial density in the sample. The H & E staining showed 70% to 98% of sensitivity and specificity of 89% to 98% in the identification of HP and Giemsa sensitivity of 64% to 96% and specificity of 98% to 100%^18^. HP is distributed irregularly in gastric mucous epithelial surface and the relatively low density of bacteria in various groups of patients can lead to false-negative results in methods of biopsy^15^.

There are few reports in the literature regarding endoscopic patterns of related HP gastritis using conventional endoscopy. In the initial works in 1995, some authors concluded that it was not possible to establish this diagnosis based on only endoscopy^4^ ^,^ ^28^. However, newer technologies such as magnification and chromoendoscopy^3^ ^,^ ^27^ showed that there endoscopic aspects that are clearly associated with HP infection, while others relate to uninfected or eradication^11^. Yagi et al.^32^ ^,^ ^33^ described the characteristics of endoscopic findings with magnification in the gastric body with normal appearance and negative HP: enanthema in tiny streaks or spots in "pinhole" aspect, which correspond to sub-epithelial capillaries and venules networks called RAC (regular arrangement of collecting venules). Anagnostopoulos et al^15^ demonstrated that enanthema Mosaic or speckled in the gastric body is more related to infection by HP, as also the enanthema in association with swelling of folds and exudate, indicating intense active inflammatory process. Enanthema in streaks or bands (gastropathy) and the appearance of tiny red spots corresponding to subepithelial venules coletantes (normal condition) are associated with the absence of infection by HP. These authors suggest that, using this technique to perform the biopsy pathology is not required^1^.

However, magnification and chromoendoscopy it is not available in most diagnostic centers, but also demand more time for execution and learning and does not seem to be practical in daily routine examinations. If specific patterns of HP related gastritis can be identified using conventional endoscopy, these standards could be applied to predict and select patients and biopsies could be directed to areas suspected of being infected by HP.

This study aims to verify the validity of the recognition of morphological patterns of gastritis associated with HP using conventional endoscopy, which would be helpful to favor the targeting of biopsies for the most affected areas.

## METHODS

This is an observational cross-sectional study approved by the ethics committee in local research. In this study was analyzed, prospectively, the endoscopic findings of 339 consecutive patients from May 27 2015 until July 10, 2015 in Endoclinic, SP, Brazil. Free and informed consent was obtained from all participants.

**FIGURE 1:**
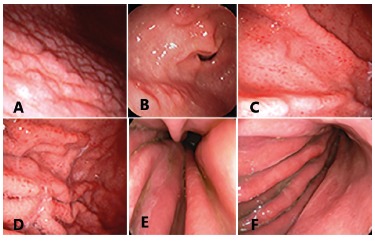
Endoscopic findings related to the positive HP: A) antral nodularity; B) raised erosions; C and D) gastric body with spotty redness or mosaic mucosal pattern; E and F) diffuse erythema with edema of gastric folds and thin layer of exudate.

**FIGURE 2:**
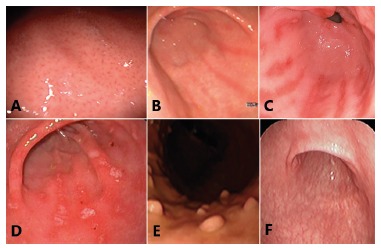
Endoscopic findings related to negative HP: A) normal appearance of the gastric body with regular arrangement of collecting venules; B) erythema in streaks or bands (red streaks); C) red streaks with flat erosions; D) flat erosions in the antrum; E) fundic gland polyps; F) extensive mucosal atrophy.

The included underwent endoscopy unit with Fujinon Pentax EPM 4400 or 3500 and all tests performed by a single professional. Sedation was carried out with 25 to 50 ug fentanolamina and midazolam 2-5 mg^19^. Images were recorded and saved in the database (OCRAM(r) system, SP) being obtained of 12-20 images per patient in all cases. These were selected for this review 6-8 images. Endoscopic aspects that were evaluated were: normal mucosa appearance, nonspecific diffuse erythema of antrum and body, erythema in streaks or bands (red streaks), mosaic mucosal pattern in the gastric body, flat erosions (minor surface defects 5 mm and flat edges), elevated (raised) erosions, nodularity of the mucosa and fundic gland polyps (Figures 1 and 2).

The diagnosis of H. pylori infection was done by the urease tests (Uretest Renylab(r), MG) performed with at least two fragments from the antrum and two from the body. The positive histological fragments were subjected to histological examination by hematoxylin and eosin (HE) staining and Giemsa stain to identify the HP, made by a pathologist who was blinded to the other results.

The following exclusion criteria were: patients with anemia, liver cirrhosis, gastric cancer, gastrectomy, renal failure, congestive heart failure, recent use of antiinflammatory drugs, aspirin, antithrombotics, use of proton pump inhibitors or H2-receptor antagonists in past two months, prior history of eradication of HP; extensive gastric mucosal atrophy.

Applied exclusion criteria were eliminated 169 patients, leaving 170, of which 52/170 (30.5%) were positive and 118/170 (69.4%) HP negative.

 The data were studied in frequency tables and contingency being used the Fisher test and chi-square association for nominal data and Mann-Whitney and unpaired t test for association of numerical data. The significance used was 5% (p = 0.05). The calculations were made with the Graph Pad Prism version 5.0 software.

## RESULTS

The positive HP endoscopic findings are shown in Table 1 where it can be seen that the most frequent finding was erythema.

**TABLE 1:** Endoscopy findings positive for Helicobacter pylori infection (n=52)

Findings	n=52	%
Normal mucosa appearance	8	15,38
Antral nodularity	14	26,92
Mosaic pattern in the body	11	21,15
Erythema of antrum and body	30	57,69
Erythema in streaks	0	0
Flat erosions	5	9,61
Elevated (raised) erosions	8	15,38
Fundic gland polyps	0	0

In Table 2 are the frequency of the findings in the negative group.

**TABLE 2:** Endoscopy findings negative for Helicobacter pylori infection (n=118)

Findings	n=118	%
Normal mucosa appearance	79	66,94
Antral nodularity	0	0
Mosaic pattern in the body	3	2,54
Erythema of antrum and body	23	21,18
Erythema in streaks	14	11,86
Flat erosions	0	0
Elevated (raised) erosions	11	9,32
Fundic gland polyps	14	11,86

In about 8/52 (15.38%) infected patients, endoscopic examination was normal while normality was present in 79/118 (66.94%) of the uninfected. Comparing the endoscopic findings in Table 3, it is possible to note that patients with HP infection have more antral nodularity, mosaic pattern in the gastric body and redness of antrum and body.

**TABLE 3:** Comparison of endoscopic findings in HP positive and negative individuals

Variable	HP positive (n=52)	HP negative (n=118)	p	OR	CI
Age years)	15- 80Average of 41.15 ± 14.84	9,0-72,0Average of 41.14±15.45	0,99 (*)		
Gender	32 women / 26 men	72 women/ 46 men	0,45(§)		
Nodularity	14 (26,92%)	0	< 0,0001(§§)	OR=89,26	95%CI= 5.19 -153
Mosaic pattern	11 (21,15%)	3 (2,54%)	0,0002(§§)	OR= 10,28	95%CI= 2,73-38,7
Diffuse redness	30 ( 57,69 %)	25 (21,18 %)	< 0,0001(§)	OR= 5,07	95%CI=2,50-10,27
Flat erosions	5 (9,61%)	14 (11,86%)	0,33(§)		
Raised erosions	8 (15,38%)	0	0.008§§)	OR=21.9	95%CI=1,16-416
Red streaks	0	11 (9,32%)	0,01 (§§)	OR= 0,089	95%CI=0,005-1,54
Polyps	0	14 (11,86%)	0,005 (§§)	OR=0.06	95%CI=0,004 -1,17

(*) = unpaired t test; (§) = chi-square test; (§§) = Fisher test

## DISCUSSION

 In the present study we sought to identify endoscopy findings related to HP infected or uninfected gastric mucosa. The selected endoscopic findings for this research have clear association with HP related gastritis and have been described in previous papers. Edema plies, with or without exudate, diffuse or patchy erythema are regarded as mucosal inflammatory process and are good indicators of the presence of HP^30^.

Atrophic gastritis areas were avoided for biopsies in this study because they are hostile regions of the mucosa to the colonization of HP, causing false-negative test results and are present in older patients with longtime HP chronic infection^7^.

Magnifying studies have shown that the numerous tiny lines or red dots in the gastric body, seen with conventional endoscopy, were regular arrangement of collecting venules (RAC), characteristic finding in normal stomach without infection by HP with 100% sensitivity and 90% especificidade^32^.

In the current study the following imaging findings showed a positive association with HP: antral nodularity, mosaic pattern in the body, diffuse redness and raised erosion.

According to the literature, the antral nodularity is significantly associated with chronic active gastritis and follicular gastritis and showed high specificity (98.5%) and high positive predictive value (91.7%) but low sensitivity (32%) for the diagnosis of HP^4^ ^,^ ^17^. In this research those images shown to be 89.2 times more frequent in infected individuals (OR = 89.26 and 95% CI = 5.19 -153) demonstrating that this finding is valuable in the diagnosis of HP infection and the endoscopic finding that best showed this association.

The raised erosions are mucosal elevations on gastric folds of the antrum and distal body containing fibrin exudation and sometimes hematin. Denote chronic inflammation and besides being frequent in patients with HP, appear also in individuals with chronic use of antiinflammatory drugs^2^. In this research the findings were nearly 22 times more common in infected patients and none in the negative group (p = 0.0081, OR = 21, 99; 95% CI = 1.16 to 416.6).

The flat erosions are mucosal continuity solutions, associated with erythema, fibrin and sometimes hematin. In general it is smaller than 5 mm in diameter and less than 1 mm depth^2^. These images were not useful for the diagnosis in question. They represented 9.61% of HP positive group and 11.86% of HP negative group, with p = 0.33.

The redness of the mucosa was the most common finding. For this search, this kind of image was divided into diffuse redness in antrum and body, red strikes (according to the literature is found most negative HP cases) and mosaic pattern (more related in the HP positive cases)^30^. In this study diffuse redness was found in 30 patients in positive group HP (57.69%) and in negative group 25 (21.18%), being 5.7 times more common in infected individuals (p <0.0001, OR = 5.07, 95% CI 2.50 to 10.27). The mosaic pattern was found in 11 patients in positive group (21.15%) and in only three patients (2.54%) in the negative group (p = 0.0002; OR = 10.28; 95% CI 2.73 to 38.7 in).

Although red streaks findings have shown negative association with infection, this association could not be confirmed by analyzing the confidence interval obtained. On the negative HP group were found 11 patients with this aspect (9.32%) and none in HP positive group (p = 0.01; OR = 0.089 95% CI = 0.005 to 1.54).

Fundic gland polyps, according to literature^10^ ^,^ ^29^ are associated only with uninfected cases. In this work all detected polyps are fundic gland and in all cases HP was negative (n = 14; 11.86%). No hyperplastic polyp (associated with the presence of HP and with congestive gastropathy) was found and also no adenomatous polyp (associated with intestinal metaplasia).

Polyps fundic gland, red streaks and normal mucosal appearance correlate with the negativity of HP infection as other studies^7^ ^,^ ^10^ ^,^ ^29^, but such associations could not be here demonstrated when analyzing the confidence interval obtained. With the selective collection of fragments for histological study in the supposedly positive cases^5^ ^,^ ^6^ ^,^ ^9^ ^,^ ^12^ it avoids the routine submission for pathology in cases of morphological patterns not related to infection and with HP negative urease test.

## CONCLUSION

Endoscopic findings are useful predictability of location and direction of biopsies in the HP research. The most representative form of HP related gastritis was the nodularity of the antral mucosa. The raised erosion and mucosa in mosaic in the body are suggestive but not specific to the infection. The other forms were not conclusive of the presence of HP.

## References

[1] Anagnostopoulos GK, Yao K, Kaye P, Fogden E, Fortun P, Shonde A (2007). High-resolution magnification endoscopy can reliably identify normal
gastric mucosa, Helicobacter pylori associated gastritis, and gastric
atrophy. Endoscopy.

[2] Appelman HD (1994). Gastritis: terminology, etiology, and clinicopathological
correlations: another biased view. Hum Pathol.

[3] Assirati FS, Hashimoto CL, Dib RA, Fontes LHS, Navarro-Rodriguez T (2014). High definition endoscopy and "narrow band imaging" in the diagnosis
of gastroesophageal reflux disease. Arq Bras Cir Dig.

[4] Bah A, Saraga E, Armstrong D, Vouillamoz D, Dorta G, Duroux P (1995). Endoscopic features of Helicobacter pylori-related
gastritis. Endoscopy.

[5] Bahú M G, Silveira TR, Maguilnick I, Ulbrich-Kulczynski J (2003). Endoscopic nodular gastritis an endoscopic indicator of high-grade
bacterial colonization and severe gastritis in children with Helicobacter
pylori. J Pediatr Gastroenterol Nutr.

[6] Calabrese C, Di Febo G, Brandi G, Morselli-Labate AM, Areni A, Scialpi C (1999). Correlation between endoscopic features of gastric antrum, histology
and Helicobacter pylori infection in adults. Ital J Gastroenterol Hepatol.

[7] Cho JH, Chang YW, Jang JY, Shim JJ, Lee CK, Dong SH (2013). Close observation of gastric mucosal pattern by standard endoscopy can
predict Helicobacter pylori infection status. J Gastroenterol Hepatol.

[8] Dixon MF, Genta RM, Yardley JH, Correa P (1996). Classification and Grading of Gastritis: The Updated Sydney System in
the International Workshop on the Histopathology of Gastritis, Houston
1994. American Journal of Surgical Pathology.

[9] Magalhães MAB, Barbosa AJA, Figueiredo JA, Alberti LR, Petroianu A (2015). Effects of different periods of gastric ischaemia in the viability of
the tissue of body, fundus and antrum region of rabbit stomach. Arq Bras Cir Dig.

[10] Elhanafi S, Saadi M, Lou W, Mallawaarachchi I, Dwivedi A, Zuckerman M (2015). Gastric polyps Association with Helicobacter pylori status and the
pathology of the surrounding mucosa, a cross sectional study. World J Gastrointest Endosc.

[11] Gonen C, Simsek I, Sarioglu S, Akpinar H (2009). Comparison of high resolution magnifying endoscopy and standard
videoendoscopy for the diagnosis of Helicobacter pylori gastritis in routine
clinical practice a prospective study. Helicobacter.

[12] Henry MACA (2014). Diagnosis and management of gastroesophageal reflux
disease. Arq Bras Cir Dig.

[13] Ji R, Li YQ (2014). Diagnosing Helicobacter pylori infection in vivo by novel endoscopic
techniques. World J Gastroenterol.

[14] Kato T, Yagi N, Kamada T, Shimbo T, Watanabe H, Ida K (2013). Diagnosis of Helicobacter pylori infection in gastric mucosa by
endoscopic features a multicenter prospective study. Dig Endosc.

[15] Khulusi S, Mendall MA, Patal P, Levy J, Badve S, Badve S (1995). Helicobacter pylori infection density and gastric inflammation in
duodenal ulcer and non-ulcer subjects. Gut.

[16] Komoto K, Haruma K, Kamada T, Tanaka S, Yoshihara M, Sumii K (1998). Helicobacter pylori infection and gastric neoplasia correlations with
histological gastritis and tumor histology. Am J Gastroenterol.

[17] Laine L, Cohen H, Sloane R, Marin-Sorensen M, Weinstein WM (1995). Interobserver agreement and predictive value of endoscopic findings
for H pylori and gastritis in normal volunteers. Gastrointest Endosc.

[18] Laine L, Lewin DN, Naritoku W, Cohen H (1997). Prospective comparison of H&E, Giemsa, and Genta stains for the
diagnosis of Helicobacter pylori. Gastrointestinal Endoscopy.

[19] Leslie K, Stonell CA (2005). Anesthesia and sedation for gastrointestinal Endoscopy. Curr Opin Anaesthesiol,.

[20] Loffeld RJ (1999). Diagnostic value of endoscopic signs of gastritis with special
emphasis to nodular antritis. Neth J Med.

[21] Lopes AI, Vale FF, Oleastro M (2014). Helicobacter pylori infection - recent developments in diagnosis
World. J Gastroenterol.

[22] Malfertheiner P, Mégraud F, O'Morain C, Hungin AP, Jones R, Axon A (2002). Current concepts in the management of Helicobacter pylori
infection-the Maastricht 2-2000 Consensus Report. Aliment Pharmacol Ther.

[23] Mihara M, Haruma K, Kamada T, Komoto K, Yoshihara M, Sumii K (1999). The role of endoscopic findings for the diagnosis of Helicobacter
pylori infection evaluation in a country with high prevalence of atrophic
gastritis. Helicobacter.

[24] Misra SP, Dwivedi M, Misra V, Agarwal SK, Gupta R, Gupta SC (1990). Endoscopic and histologic appearance of the gastric mucosa in patients
with portal hypertension. Gastrointest Endosc.

[25] Morais M, Macedo EP, Silva MR, Rohr MRS, Ferraz MLG, Castro RRO (1997). Comparação entre testes invasivos para o diagnóstico da infecção pelo
Helicobacter pylori. Arq Gastroenterol.

[26] Ornellas L C, Cury MS, Lima VM, Ferrari AP (2000). Avaliação do teste rápido da urease conservado em
geladeira. Arq. Gastroenterol.

[27] Ratin ACF, Orso IRB (2015). Minimal endoscopic changes in non-erosive reflux
disease. Arq Bras Cir Dig.

[28] Redéen S, Petersson F, Jönsson KA, Borch K (2003). Relationship of gastroscopic features to histological findings in
gastritis and Helicobacter pylori infection in a general population
sample. Endoscopy.

[29] Sakai N, Tatsuta M, Hirasawa R, Iishi H, Baba M, Yokota Y (1998). Prevalence of Helicobacter pylori infection in patients with
hamartomatous fundic polyps. Dig Dis Sci.

[30] Watanabe K, Nagata N, Nakashima R, Furuhata E, Shimbo T, Kobayakawa M (2013). Predictive findings for Helicobacter pyloriuninfected, -infected and
-eradicated gastric mucosa Validation study. World J Gastroenterol.

[31] Watanabe M, Kato J, Inoue I, Yoshimura N, Yoshida T, Mukoubayashi C (2012). Development of gastric cancer in nonatrophic stomach with highly
active inflammation identified by serum levels of pepsinogen and Helicobacter
pylori antibody together with endoscopic rugal hyperplastic
gastritis. Int J Cancer.

[32] Yagi K, Nakamura A, Sekine A (2002). Characteristic endoscopic and magnified endoscopic findings in the
normal stomach without Helicobacter pylori infection. J Gastroenterol Hepatol.

[33] Yagi K, Nakamura A, Sekine A (2002). Comparison between magnifying endoscopy and histological, culture and
urease test findings from the gastric mucosa of the corpus. Endoscopy.

[34] Yan SL, Wu ST, Chen CH, Hung YH, Yang TH, Pang VS (2010). Mucosal patterns of Helicobacter pylori-related gastritis without
atrophy in the gastric corpus using standard endoscopy. World J Gastroenterol.

[35] Zerbib F, Vialette G, Cayla R, Rudelli A, Sauvet P, Bechade D (1993). Follicular gastritis in adults Relations with Helicobacter pylori,
histological and endoscopic aspects. Gastroenterol Clin Biol.

